# Applying explainable artificial intelligence methods to models for diagnosing personal traits and cognitive abilities by social network data

**DOI:** 10.1038/s41598-024-56080-8

**Published:** 2024-03-04

**Authors:** Anastasia S. Panfilova, Denis Yu. Turdakov

**Affiliations:** 1 Institute of Psychology of the Russian Academy of Science, Laboratory of Psychology and Psychophysiology of Creativity, Moscow, Russia; 2 Ivannikov Institute for System Programming of the Russian Academy of Science, Research Center for Trusted Artificial Intelligence, Moscow, Russia

**Keywords:** Psychology, Computer science, Statistics

## Abstract

This study utilizes advanced artificial intelligence techniques to analyze the social media behavior of 1358 users on VK, the largest Russian online social networking service. The analysis comprises 753,252 posts and reposts, combined with Big Five personality traits test results, as well as assessments of verbal and fluid intelligence. The objective of this research is to understand the manifestation of psychological attributes in social media users' behavior and determine their implications on user-interaction models. We employ the integrated gradients method to identify the most influential feature groups. The partial dependence plot technique aids in understanding how these features function across varying severity degrees of the predicted trait. To evaluate feature stability within the models, we cluster calculated Shapley values. Our findings suggest that the emotional tone (joy, surprise, anger, fear) of posts significantly influences the prediction of three personality traits: Extraversion, Agreeableness, and Openness to Experience. Additionally, user social engagement metrics (such as friend count, subscribers, likes, views, and comments) correlate directly with the predicted level of Logical thinking. We also observe a trend towards provocative and socially reprehensible content among users with high Neuroticism levels. The theme of religion demonstrates a multidirectional relationship with Consciousness and Agreeableness. Further findings, including an analysis of post frequency and key text characteristics, are also discussed, contributing to our understanding of the complex interplay between social media behavior and psychological traits. The study proposes a transition from the analysis of correlations between psychological (cognitive) traits to the analysis of indicators of behavior in a social network that are significant for diagnostic models of the corresponding traits.

## Introduction

The endeavor to diagnose psychological personality traits by evaluating long-term human behavior represents a significant innovation. Traditional methods such as laboratory experiments, social experiments, psychodiagnostics, and advanced imaging techniques like positron emission tomography and functional magnetic resonance imaging, primarily examine data acquired within a relatively brief timeframe. These methodologies are limited in their ability to assess human behavior over extended periods. As a result, how these evaluated traits manifest in an individual's immediate behavior remains largely unexplored.

In particular, the personality correlates of Facebook users' self-esteem and some of the ways in which personality traits are expressed when acquiring information have been examined^1^. The study has found a number of links between personality and behavior on Facebook. The personality trait such as Extraversion predicted not only self-reported frequency of social network use, but also the time of stay on the site. Consistent with high levels of socialization in real life, extroverts also seek virtual social contacts and are more engaged during online interactions than introverts. On social networks, this interaction leaves a noticeable behavioral trace in the form of friend lists, image posts, etc. Thus, social media exists not as an escape from reality, but as an essential tool for understanding patterns in human culture and behaviour, exposing stereotypes inherent in our everyday lives^1^.

It has been shown that in online and offline contexts (i.e. behavior on a social network and behavior in everyday life), these personality measures are invariant, although the level of traits expression in them varies and is generally lower in the online environment^2^. The greatest level of difference between contexts was revealed for Conscientiousness and Extraversion, which is discussed as a contextual influence of the cultural type associated with norms of psychological expression (ibid.). The peculiarity of the online context in comparison with the offline context may lie in the lower demand for self-control, interpersonal skills and empathy, as well as in the peculiar adjustment of the user’s social circle to his psychological characteristics, values, and interests. Thus, social media somewhat limits the expression of the Big Five.

The relationship between Extraversion and social networking behavior is presented in a systematic review of 182 studies by Bowden-Green^3^. Convincing evidence is presented that extroverts are more inclined to use social networks, use them more often, and spend more time on them. Moreover, they regularly create content using “social” vocabulary and addressing social processes. Also, extroverts often comment, give feedback to others, and share content publicly. The attitudes expressed by extroverts indicate that they value social media and are motivated to use it to achieve their social goals. Consistent with this picture, the result of a US sample (N = 576) confirmed that there was a significant positive relationship between Facebook use and the traits Extraversion and Agreeableness^4^.

A systematic review of 159 studies on the relationship between Neuroticism and social media behavior was conducted by Bowden-Green^5^. The following main research topics were identified: (1) aggression, trolling, excessive use of social networks, (2) specific unintentional patterns of network use that carry information about the user’s Neuroticism, available to his friends and subscribers, (3) creation of specific content, arbitrary, in connection with Neuroticism (self-presentation, status updates, photos), (4) reactions to content in connection with Neuroticism (likes, comments), (5) user profile characteristics (for example, network size), (6) perception of social media (motivation, profile privacy). Within the framework of these topics, the following conclusions were obtained. There is no convincing evidence that people with high Neuroticism use social networks more often than others and for longer periods of time, and are also more likely to show aggression. The connection between Neuroticism and the frequency of status updates, commenting on the content of other users, and likes has not been confirmed. Individuals with high Neuroticism have small networks of contacts on social networks, despite high motivation for active social interaction. They are also quite passive in their use of the social network, i.e. not particularly active in content creation.

For example, it has been shown that more active users of social networks are characterized by greater Openness to experience, Conscientiousness, and Extraversion in an online context, while in an offline context they exhibit these traits to a lesser extent^2^. This leads to the building of explanatory constructs about social compensation in a social network, for example, among introverts (poor-get-richer or social compensation effects of social media)^6^. A meta-analysis by Azucar et al. aimed to examine the predictive power of specific types of digital footprints on social media in predicting the Big Five traits^7^. The meta-analysis yielded the following correlation coefficients with digital traces: 0.39 for Openness to experience, 0.35 for Conscientiousness, 0.40 for Extraversion, 0.29 for Agreeableness, and 0.33 for Neuroticism. There were no differences detected in effect sizes between traits. In addition to digital footprints, the use of demographic information improved the prediction of Agreeableness and Neuroticism (trending for Openness to experience). Similar results were obtained in a meta-analysis by D. Marengo and C. Montag (Marengo, Montag, 2020): on average, the accuracy of predicting personality traits was moderate (r = 0.3), while its accuracy increased when demographic characteristics and several types of digital traces were included in the model. Among the traits, Extraversion had the greatest predictive accuracy (r = 0.39), and Agreeableness had the least accuracy (r = 0.28).

The rapid evolution of Big Data analytics^8^ and Machine Learning (ML) methods, including Deep Learning^9,10^, now offers the potential to construct mathematical models that predict psychological properties based on long-term data. This approach offers an unobtrusive observation of individuals, who are often oblivious to the continuous evaluation of their actions from a psychological perspective. An example of a more modern approach is the use of artificial neural network capabilities to build a predictive model for the Big Five traits based on data about Facebook users and their activity (N = 7438 from the myPersonality database)^11^. Facebook activity markers included posting, tagging, group membership, number of likes, events, updates, and a range of demographic characteristics. The applied model showed encouraging results, in 85% of cases accurately classifying network users based only on their activity data. An accuracy of 82.2% for predicting the Big Five traits was obtained based only on Facebook profile characteristics of users, even without taking into account their gender and age^12^. The list of online behavior markers included: likes, favorites, language, books, work, education, sports, activities, games, groups, movies, music, subscriptions, friends, interests and hobbies, links, television programs, questions, posts, number of texts and photos in the feed, number of photos without texts, news feed, etc. The selection of precisely such digital traces was made by the authors in connection with the assumption that it is social activity and social interactions that are most indicative of the manifestation of personality in social media.

For example, Map Reduce Back Propagation Neural Networks find accuracies of 91.40%, 93.89%, 91.33%, 90.43%, and 89.13% for Conscientiousness, Openness to experience, Extraversion, Neuroticism, and Agreeableness, respectively^13^. The Support Vector Machine based classifier achieves an accuracy of 87.5%^14^. The model on XGBoost has also been shown to demonstrate accuracy in the range of 65–78% for the Big Five traits considered^15^.

The works of researchers concerning the Russian social network VKontakte show the relationship between personal characteristics and the activity characteristics of users of social networks. In the article by A.B. Goncharov and I.M. Azhmukhamedov presents the identified correlations between personal characteristics and words that are most often found in posts liked by users. The identified correlations were used as a training sample to build a regression model to predict the user’s personal characteristics^16^.

Vaid and Harari describe the results of a study in which participants have been surveyed regarding the frequency of use of various social networks and platforms in their relationship with personality traits^17^. The authors have found that Openness and Extraversion are associated with the use of Facebook and messaging platforms. Neuroticism is also associated with the use of image-sharing and microblogging platforms. The authors note differences in some of the results obtained due to the country in which the sample was collected. Vaid S. and Harari G. have found that Conscientiousness is associated with the use of media sharing platforms, supporting past research linking Conscientiousness with increased use of the YouTube platform^18^. Bayer's study, like that of Vaid S. and Harari G., demonstrates the need to differentiate the specific types of social media use being studied to better understand the social media ecology of individual users and the platform features that may drive their use^19^.

However, it is crucial to note that as social norms evolve, so too does socially accepted behavior. This evolution can result in the accentuated display of specific personality traits, which can vary significantly in intensity between individuals. In recent years, several studies have applied Deep Learning methods to predict the severity of psychological traits by analyzing human behavior. Notably, corporations like Amazon and Uber have leveraged such models in their hiring processes^20–23^, considering candidates' behavior on social media^12,15,24–29^, including meta-analyses^7,30^, digital traces^31–35^, acoustic speech analysis^36,37^, dynamics of facial emotional expressions^38^.

With the increasing utilization of these models comes the challenge of trust and interpretability of the results generated. This is where Explainable Artificial Intelligence (XAI) methods come into play, facilitating a deeper understanding of the models' inner workings^39,40^. These methods are increasingly finding application in diagnosing psychological traits and various states of personality through behavior analysis^41^.

In mental health research, the demand for explainability and interpretability is on the rise^42–44^, particularly since data describing syndromes, outcomes, disorders, and symptoms often interrelate probabilistically, as evidenced in studies analyzing suicide predictors^45,46^. XAI methods also serve to detect and mitigate various types of bias introduced by the characteristics of the samples or the algorithms utilized^47^, thereby enhancing model performance^48^.

Our research focuses on understanding the manifestation of psychological traits and cognitive abilities in user behavior on VK^49^, a popular social networking platform. The relevance of the study is conditioned by the fact that, on the one hand, the publications describe significant differences in the manifestation of certain properties depending on the online platform and the country where the sample has been collected, on the other hand, the publications do not contain any analysis of the largest Russian social network VKontakte and the corresponding sample. Also, existing research is based on correlation analysis, analysis of the influence of features in machine learning models that contain this information, but the publications do not present an analysis of deep learning models from the point of view of explainable artificial intelligence methods, which makes it possible to assess more accurately the performance of features and represents the novelty of this research. For analysis, we have identified categories of features that characterize both the user himself and his activity indicators on the social network and the characteristics of his posts, which are subjected to further analysis. As part of this study, we want to obtain answers to the following questions: (1) What is the strength of influence of various categories of attributes (indicators of user activity, personal characteristics, subject matter and emotional coloring of posts, frequency of posts) in trained neural network models for predicting personality traits and intelligence? (2) What is the mechanism of influence (direct, inverse) of features categories on the prediction of neural network models regarding the degree of expression of psychological traits and level of intelligence? (3) What are the particular qualities of clustering the calculated coefficients of the features that influence the prediction of the neural network model for each sample instance. We employ ML models and XAI methods in our approach. The subsequent sections detail our sample and psychodiagnostic tools used, feature generation and selection methods from users' social media profiles, and data preprocessing. We describe our Deep Learning models based on Multilayer Perceptron (MLP) architecture and their training results in the section on modeling approaches. The application and features of XAI methods, including Shapley Additive Explanations, Partial Dependence Plots, and Integrated Gradients, are outlined in the section on Explainable AI methods. We discuss our key findings regarding the influence of user behavior signs on social network platforms in relation to psychological properties and cognitive abilities in the "Models Explainability" section. Finally, the "Discussion" section interprets the results from the perspective of psychological theory.

## Results

### Models explainability

#### Models explainability from the influence of various groups of characteristics point of view

To start we should consider the results of the study regarding the answer to question #1″ What is the strength of influence of different trait categories in trained neural network models for predicting personality traits and intelligence?”.

**Figure 1:**
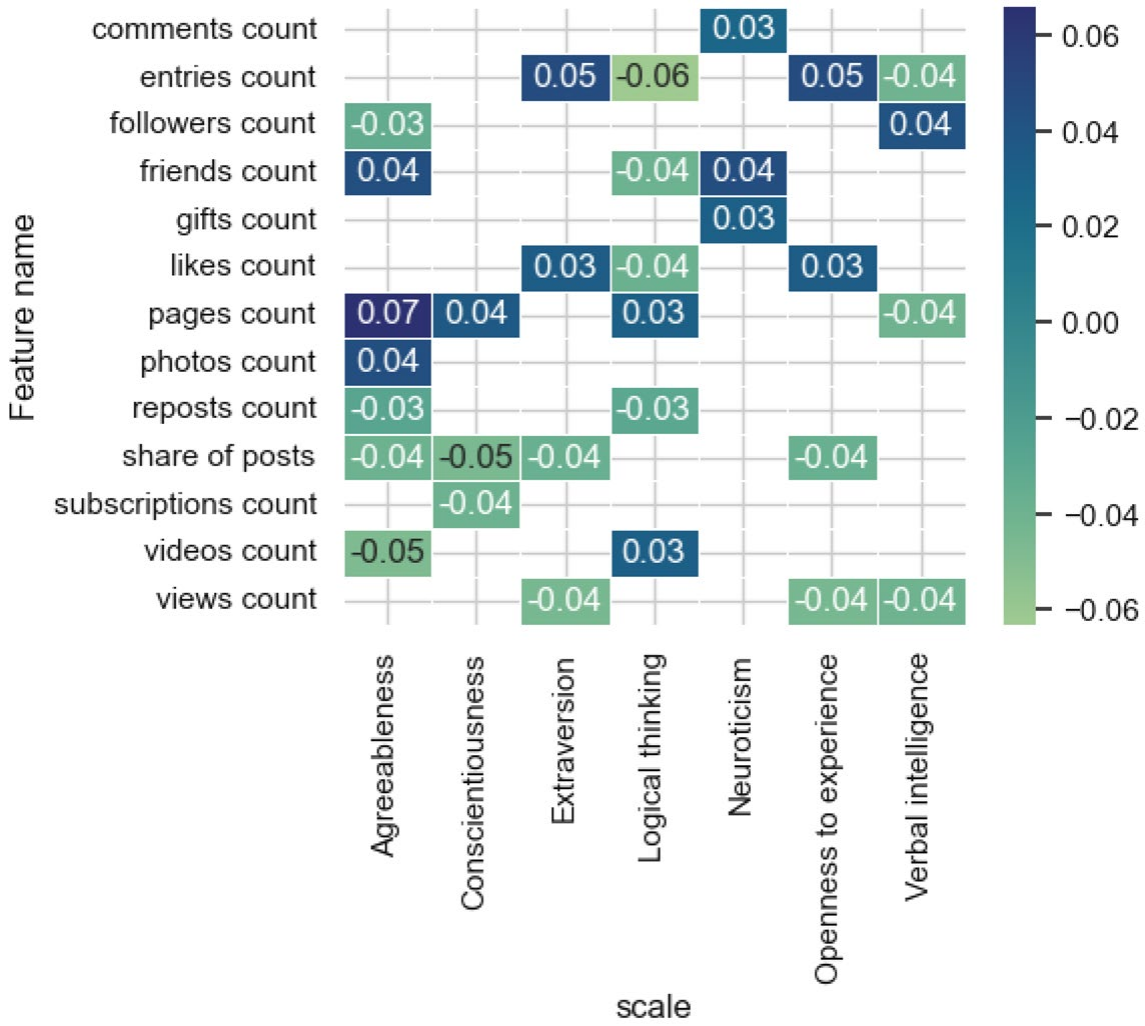
The estimates of the significance of the most influential features obtained by the integrated gradients method for the models and features category of *activity.*

To assess the influence of social network user profile characteristics on models predicting scores for each of the Big Five test scales^50^, level of Logical thinking^51^ and verbal intelligence^52^ we applied one of the methods of explainable artificial intelligence to trained models (integrated gradient method^53^). Figure 1 presents the results of evaluating the most influential features from the features category of* activity.*

When considering this result, the direction of influence (positive or negative) does not matter; attention is paid to the coefficients of the signs. It can be noted that this group of characteristics is most represented in models for diagnosing Agreeableness and Logical thinking. Among the most “popular” features posts count, pages count, share of posts stand out. Thus, posts count is a more significant characteristic for predicting the level of Logical thinking, pages count for Agreeableness, and the share of posts for Conscientiousness. The model for predicting the degree of severity on the Neuroticism scale depends less on activity signs than the other considered models.

**Figure 2:**
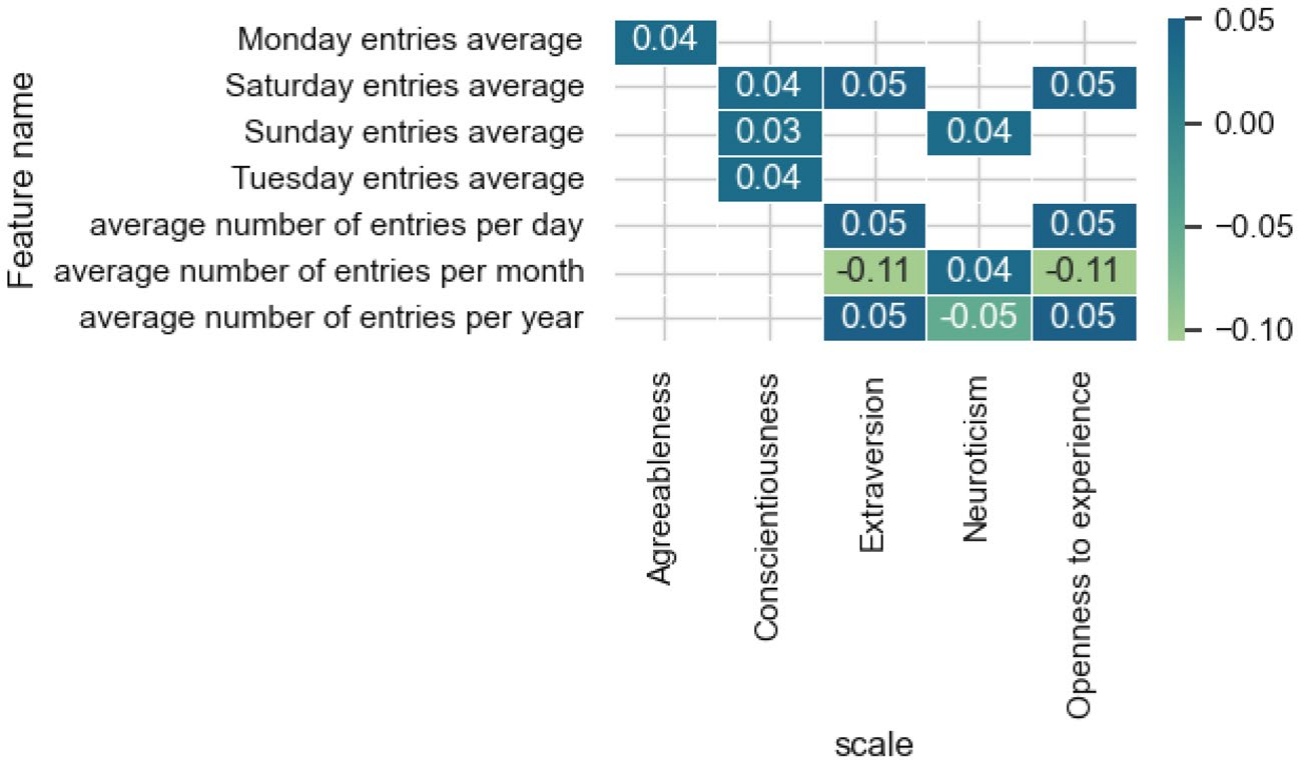
The estimates of the significance of the most influential features obtained by the integrated gradients method for the models and features category of *frequency.*

Figure 2 presents the results of evaluating the most influential features from the features category of *frequency*.

Among the most influential traits is the average number of posts per month, which is significant for the model predicting the level of Extraversion and Openness to experience. Also, the models for these two personality traits are more dependent on the post frequency category. Among the signs of the frequency of posting posts on a social network by day of the week, Saturday and Sunday stand out, which are predominantly weekend days of the week. In intelligence diagnostic models, this category of traits does not have a significant effect.

**Figure 3:**
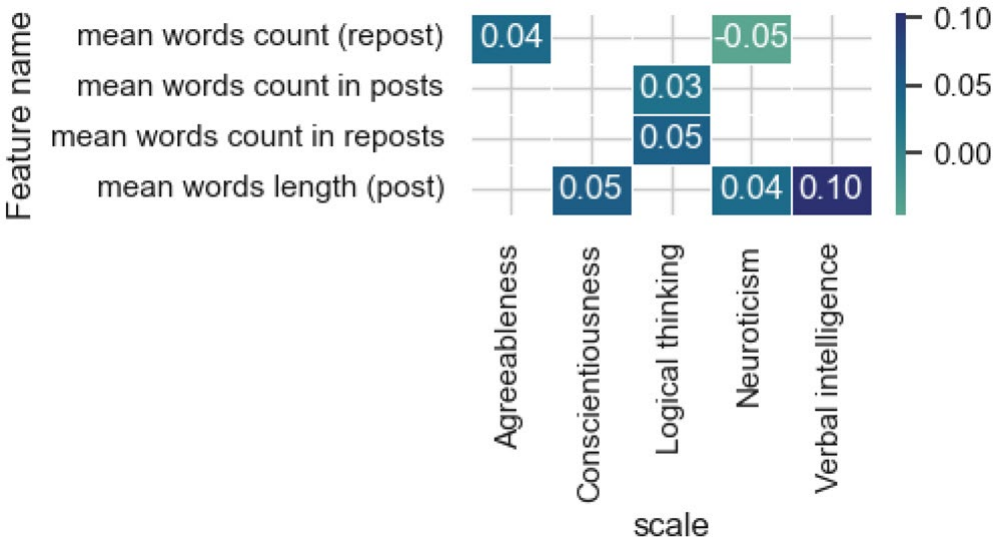
The estimates of the significance of the most influential features obtained by the integrated gradients method for the models and features category of* text characteristics.*

Figure 3 presents the results of evaluating the most influential features from the features category of *text characteristics*.

The average length of word indicator is highlighted in posts for the model of verbal intelligence, which is quite logical and confirmed by many studies looking into written speech in its relationship with the level of verbal abilities. For a neural network model for predicting the level of Logical thinking, the signs of the number of words in posts and reposts are significant. For the scale of Neuroticism and Agreeableness, the indicator of the number of words in reposts is highlighted. In this case, highlighting individual features for posts and reposts demonstrates its effectiveness, since posting a post relates to the user’s speech production, and reposting is a broadcast or transmission of someone else’s opinion.

**Figure 4:**
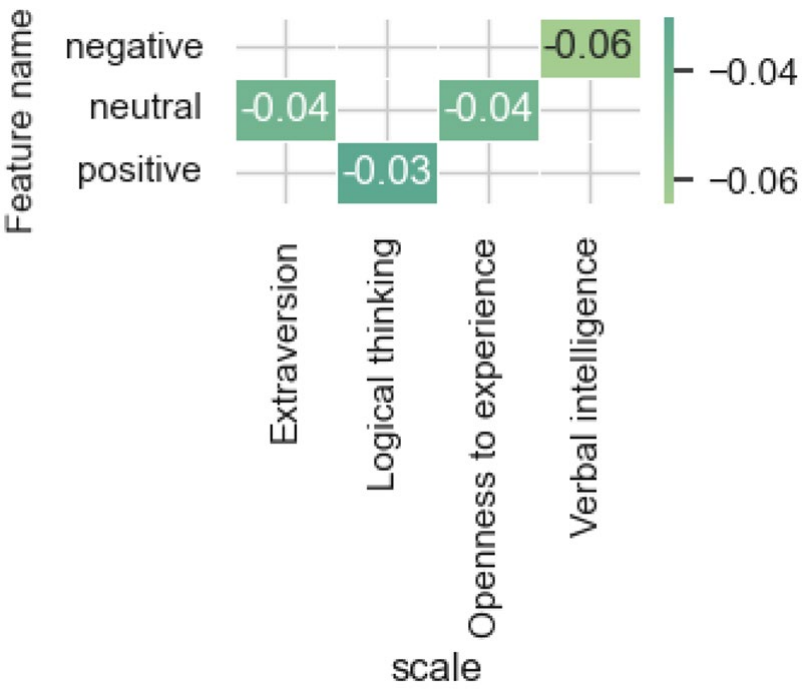
The estimates of the significance of the most influential features obtained by the integrated gradients method for the models and features category *sentiment.*

Figure 4 presents the results of evaluating the most influential features from the features category of *sentiment*.

The indicator significance of the sentiment of posts is noted for a small number of scales, among which verbal intelligence stands out for the indicator of negative sentiment. The indicator of the neutral tone of posts is significant for neural network models that predict the level of Extraversion and Openness to experience.

**Figure 5:**
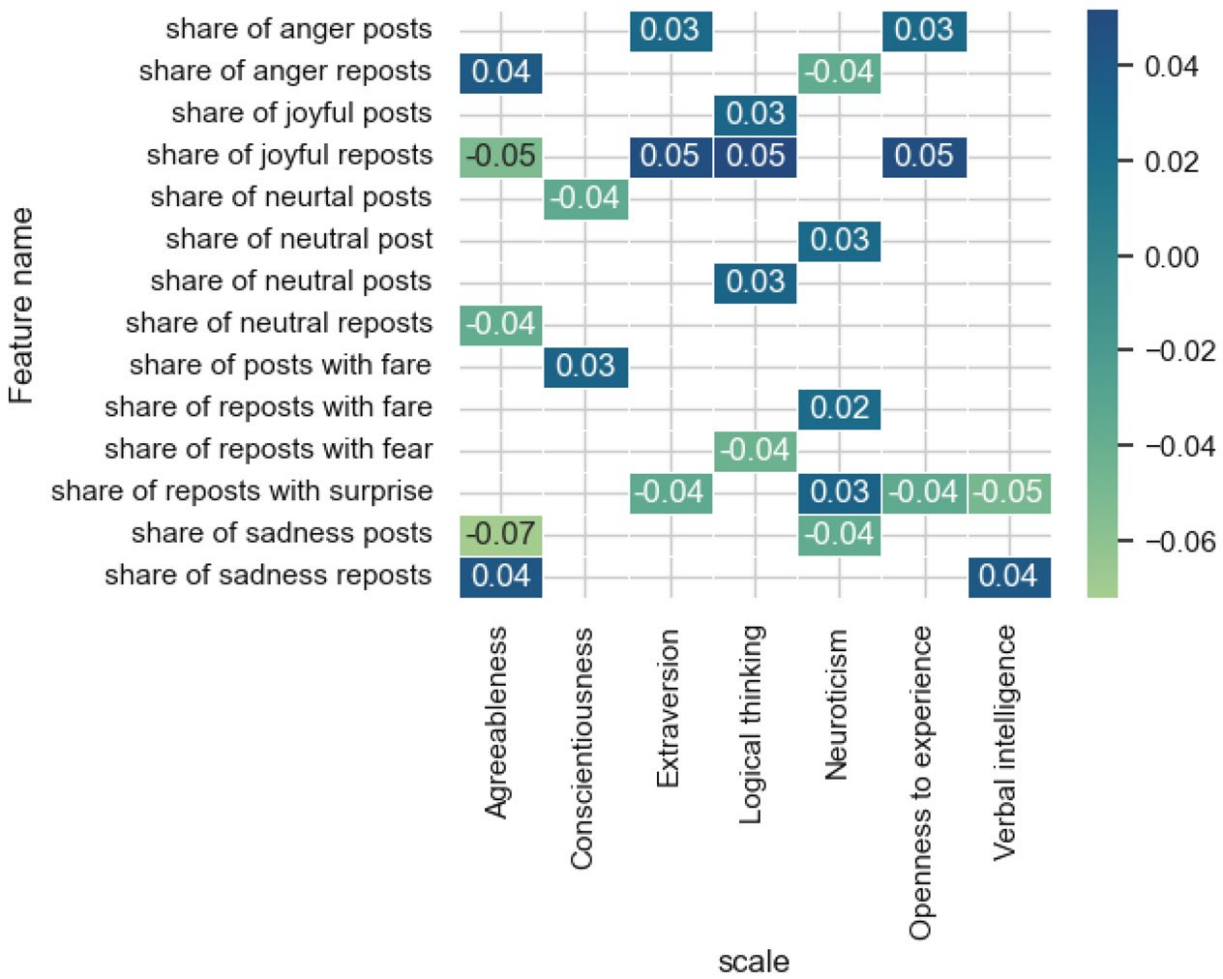
The estimates of the significance of the most influential features obtained by the integrated gradients method for the models and features category *emotional evaluation.*

Figure 5 presents the results of evaluating the most influential features from the features category of *emotional evaluation*.

The signs of the emotional characteristics of posts and reposts is significant for all the models under consideration, however, it is most represented in the models for predicting the level of Agreeableness and Neuroticism. For Agreeableness, the emotions that can be distinguished to the greatest extent are sadness (the direction of influence is not considered in this type of analysis), joy, anger and neutral emotion. For Neuroticism, the emotions of sadness, surprise, fear, anger and neutral emotion are distinguished. Emotions of joy and surprise are represented to a greater extent among the models.

**Figure 6:**
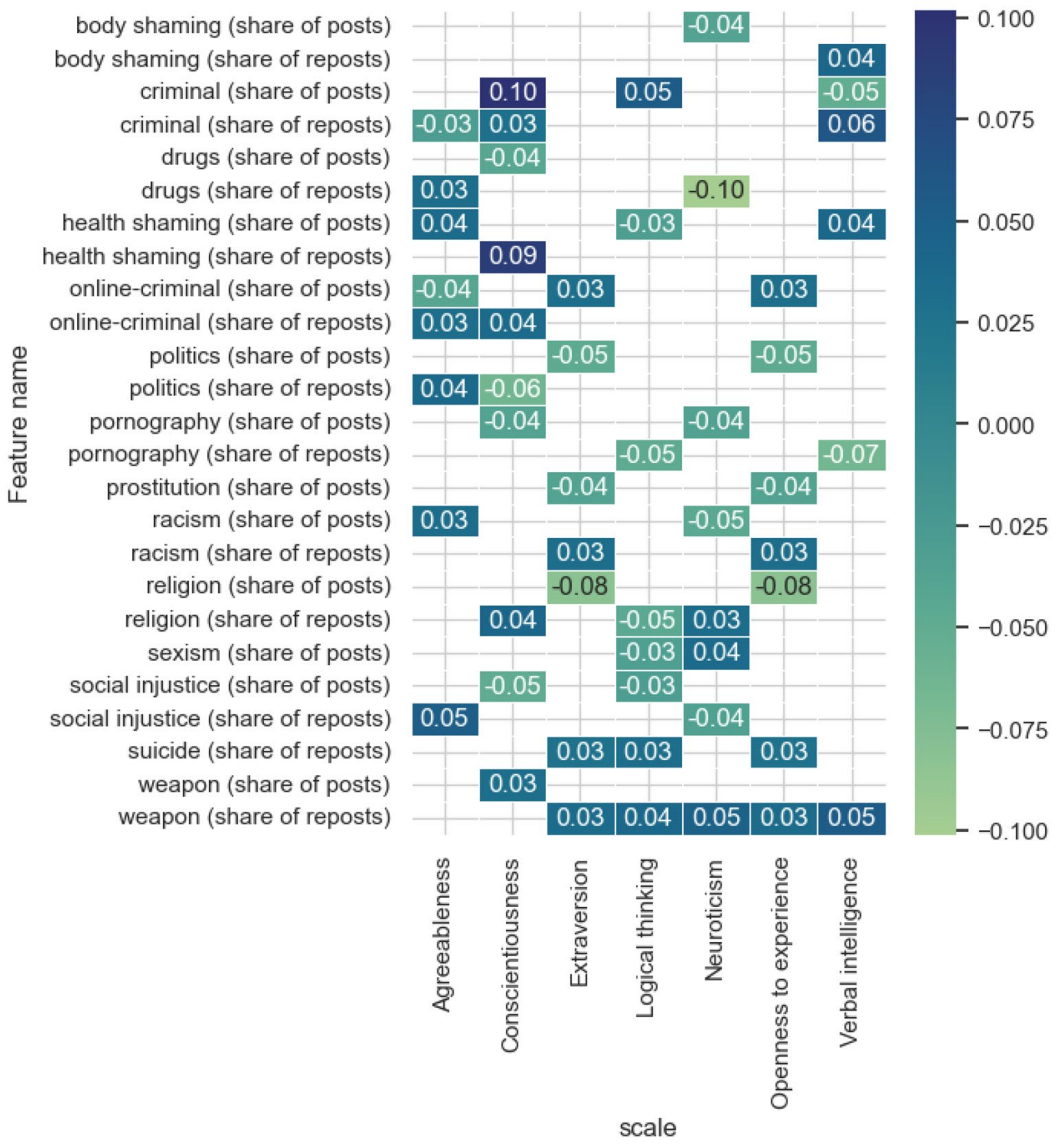
The estimates of the significance of the most influential features obtained by the integrated gradients method for the models and features category* topic.*

Figure 6 presents the results of evaluating the most influential features from the features category of *topic*.

The subject matter of the posts, as we can see from Fig. 6, is reflected for all the models under consideration. The most significant topics are crime and health shaming for the Conscientiousness scale, drugs for Neuroticism and religion for Extraversion and Openness to experience. The most significant feature in terms of the number of models is the topic of weapons. The considered category of signs in terms of their number is most represented in the model for predicting the degree of Consciousness. There are also differences in the degree of influence of features depending on the topic of the post or repost, which is also associated with expressing one’s own opinion or broadcasting someone else’s news or article or reposting it.

**Figure 7:**
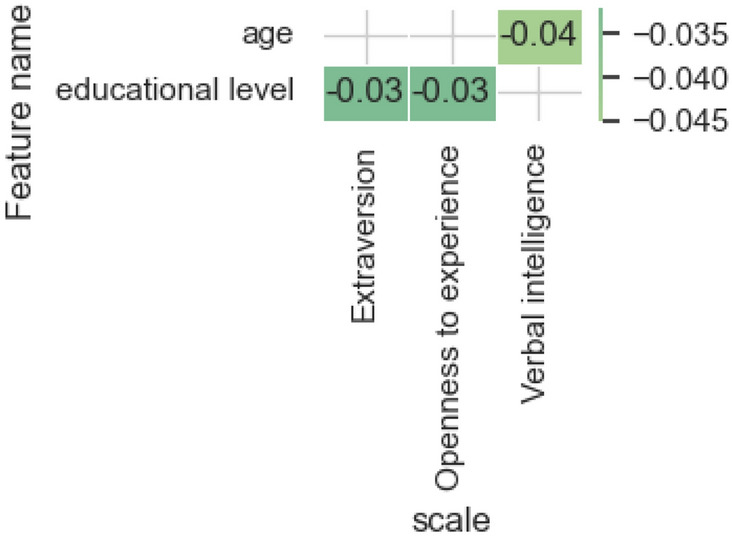
The estimates of the significance of the most influential features obtained by the integrated gradients method for the models and features category* personal characteristics.*

Figure 7 presents the results of evaluating the most influential features from the features category of *personal characteristics*.

Among the human properties under consideration, the most significant is the user’s age in its influence on the level of verbal intelligence, which is confirmed by research in the relevant field. Level of education significantly influences the model predicting the level of Extraversion and Openness to experience.

As a result of the analysis, we observe that indicators of user activity in social networks, emotional coloring of posts and topics of posts are the most universal characteristics that have a significant impact on all considered models that predict the Big Five personality traits, the level of verbal intelligence and Logical thinking. A group of signs of personal characteristics and text characteristics of posts can be called the most specific one. It can also be noted that the group of features associated with sentimental analysis of posts does not reflect all the nuances of influence compared to the group of features associated with the emotional assessment of posts.

#### Models explainability from the point of view of the signs influence mechanism

**Figure 8:**
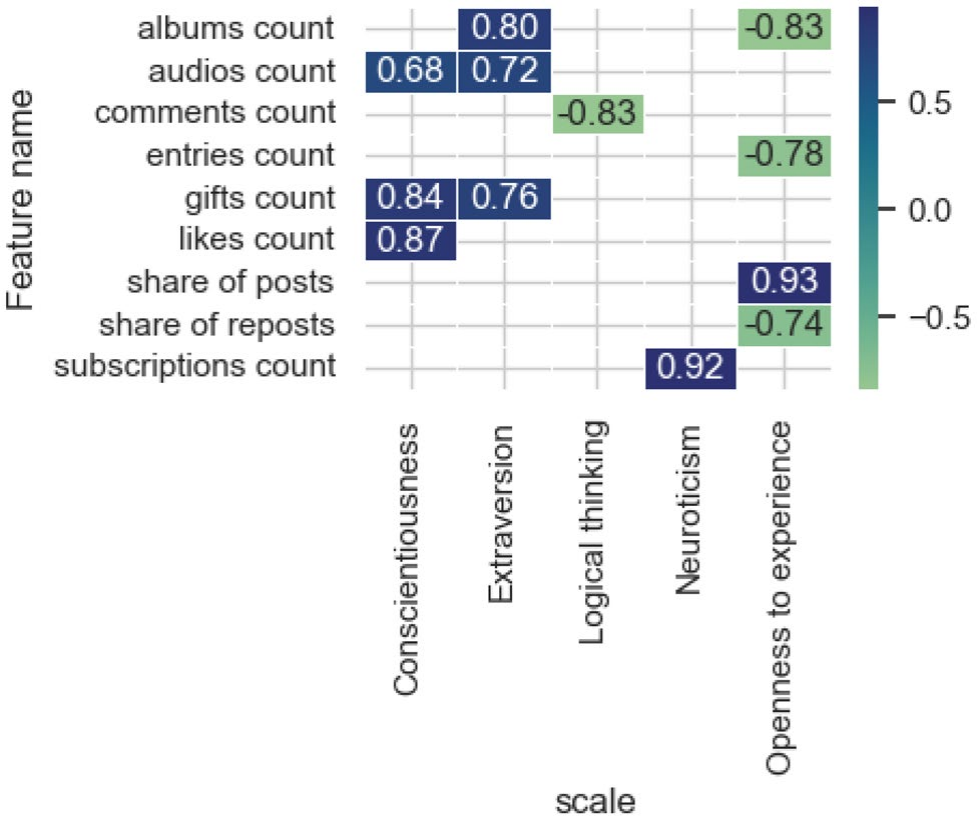
Significant correlation estimates of the signs of the activity category and the values predicted by the model on the scales.

The Partial Dependence Plot^54^ method of explainable artificial intelligence allows one to obtain estimates predicted by the model for various intervals of the characteristic. Thus, in this section we will answer question #2 “What is the mechanism of influence (direct, inverse) of features categories on the prediction of neural network models regarding the degree of expression of psychological traits and level of intelligence?” The result of the influence analysis of the attributes of various categories presented in the previous section does not answer the question about the direction of the attribute influence: positive and negative, and also does not contain information about what happens to the predicted value in connection with a change in the value of the attribute. Figure 8 shows significant correlation estimates (*p* value < 0.05) for the category of activity signs.

Among the identified significant correlation estimates, a multidirectional relationship between the proportion of posts and reposts and the score on the Openness to experience scale stands out, which indicates that participants with a high score on this scale tend to post more than repost. Extroverts typically receive a large number of gifts from other social network users, as well as listen to music and create photo albums. Users with a high level of Consciousness are characterized by a large number of likes for their materials, audio recordings and gifts. At the same time, the Logical thinking scale shows an inverse relationship with the number of comments. People with high levels of Neuroticism are characterized by a large number of subscriptions to various communities (groups). Thus, the analysis allows us to determine the motivational components of using a social network by users with different sets of properties.

**Figure 9:**
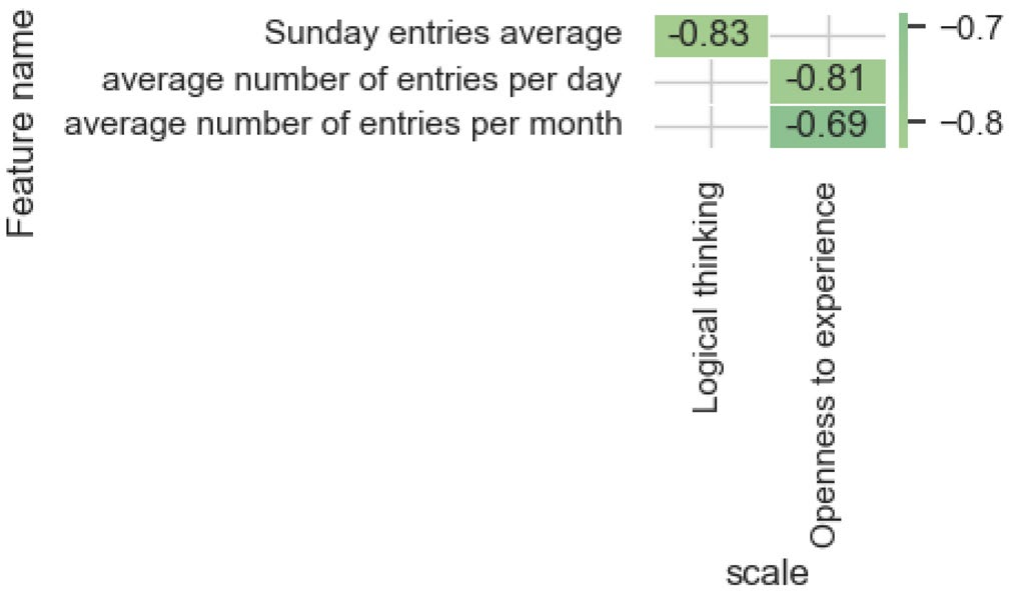
Significant correlation estimates of the characteristics of the frequency category and the values predicted by the model on the scales.

Figure 9 contains significant correlation estimates (*p* value < 0.05) for the frequency category and demonstrates an inverse relationship with the level of Logical thinking regarding posting on a weekend. Users with a high level of Openness to experience are characterized by a low frequency of publishing material per day and month, and the opposite is also true.

**Figure 10:**
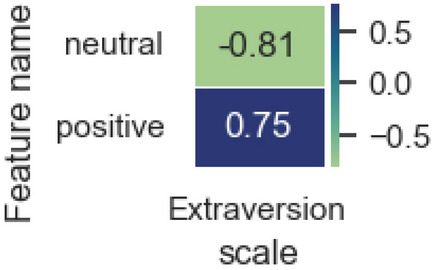
Significant correlation estimates of sentiment category attributes and model-predicted scale values.

Figure 10 contains significant correlation estimates (*p* value < 0.05) for the sentiment trait category and demonstrates a multidirectional relationship between the neutral and positive coloring of posts with the Extraversion scale, which is associated with the peculiarity of demonstrating a positive mood to people with a high score on this scale.

**Figure 11:**
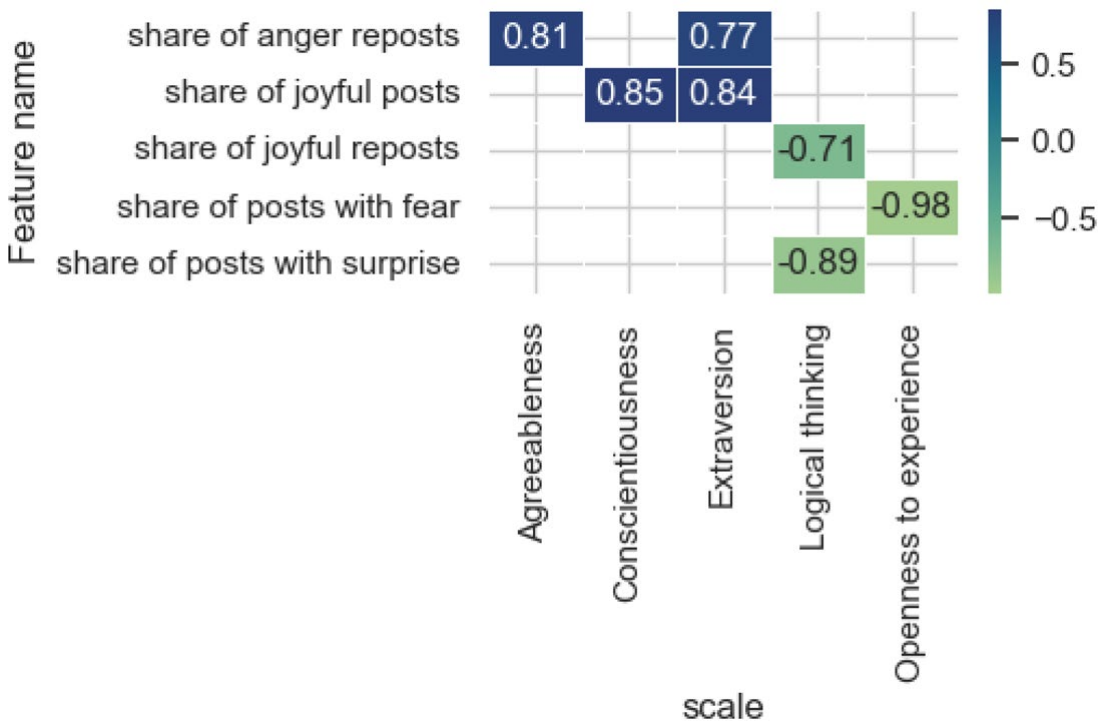
Significant correlation estimates of the attributes of the emotional evaluation category and the values predicted by the model on the scales.

Figure 11 shows significant correlation estimates (*p* value < 0.05) for the emotional evaluation feature category. There is an inverse relationship with the share of posts and reposts with emotions of joy and surprise with the level of Logical thinking. Users with a high level of Openness to experience are not likely to post a large number of posts with the emotion of fear, and the opposite is also true. Noteworthy is the direct relationship between the share of posts with the emotion of joy and the predicted values on the scale of Conscientiousness and Extraversion, as well as the share of reposts with the emotion of anger for the scales of Agreeableness and Extraversion.

**Figure 12:**
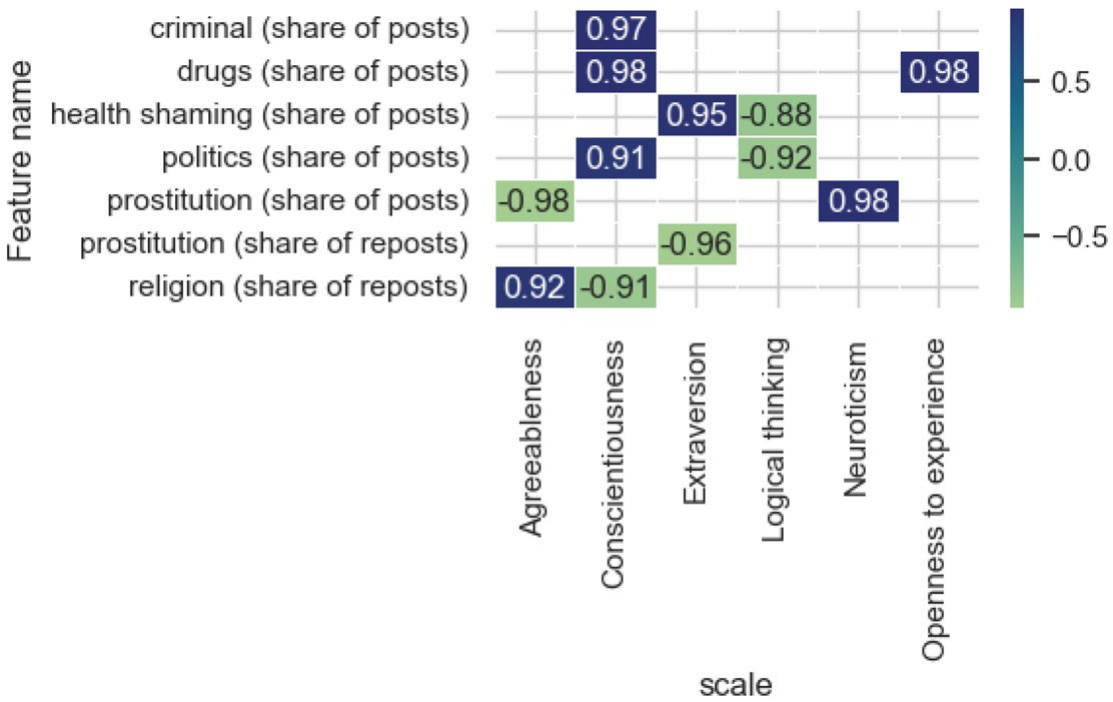
Significant correlation estimates of the topic category features and the model-predicted scale values.

Figure 12 shows significant correlation estimates (*p* value < 0.05) for the topic feature category. Noteworthy is the inverse relationship between the share of posts on the topic of health shaming and politics and the predicted level of Logical thinking. It is not typical for people with a high level of consciousness to post a large number of reposts on the topic of religion; however, there is an increase in the share of posts on the topic of crime, drugs and politics. It is worth noting that high correlation values for the proportion of posts on the topic of drugs for such inherently different psychological properties as Consciousness and Openness to new experience may mean that these posts are posted for different purposes. Thus, a person with a high degree of Openness to new experience is characterized by a desire to try something new and experiment; a conscientious person, on the contrary, can post this material in order to warn other network users about harmful consequences.

As for other groups of features (text characteristics and personal characteristics), the results of the analysis did not demonstrate significant correlations with the studied properties, which may indicate the absence of a linear relationship between them.

The discovery of significant correlations between the values of features associated with the emotional coloring of the text and tonality reflects one of the key properties of this trait, which is a vivid expression of emotions. That is, we can conclude that the way of expressing oneself is transferring from offline to online. It is not typical for people with a high level of intelligence to clearly demonstrate their emotions, which is also reflected in the results obtained, including their relationships with other network users. The lack of emotionally charged posts leads to fewer comments. Also, it is not typical for these users to waste their free time on social networks. Analysis of such results involves interpreting the obtained values through the prism of personal properties. So, for example, by posting posts with a joyful emotional coloring, an extrovert will most likely express his own state, and a conscientious person can use this to support the positive mood of his environment on a social network. People with a high level of Openness to new experiences often demonstrate a lack of fear in everyday life, having risky hobbies; we observe the transfer of this quality to behavior on a social network.

The significant features identified in the previous subsection are reflected in significant correlations.

It can be highlighted that among all the properties, the greatest consistency with the previous result on identifying significant signs is demonstrated by the property of Openness to experience in terms of signs of activity and frequency in the groups. Logical thinking was confirmed in terms of the emotional coloring of the posts and their topics. The trait of Conscientiousness demonstrated consistency across the topic category of posts. Thus, it can be noted that the application of various methods of explainable artificial intelligence to trained models can, on the one hand, confirm a number of results, increasing their reliability, and can be an additional source of information, on the other hand, which nevertheless needs to be interpreted through the essence of the assessed psychological property and its features manifestations in life.

#### Explainability of individual cases

The conducted analysis highlights the significance of assessing the influence of studied features on various psychoLogical properties and cognitive abilities. In this section, we would like to answer the 3rd question of the study “What are the particular qualities of clustering the calculated coefficients of the influence of features on the prediction of a neural network model for each sample instance?”.

**Figure 13:**
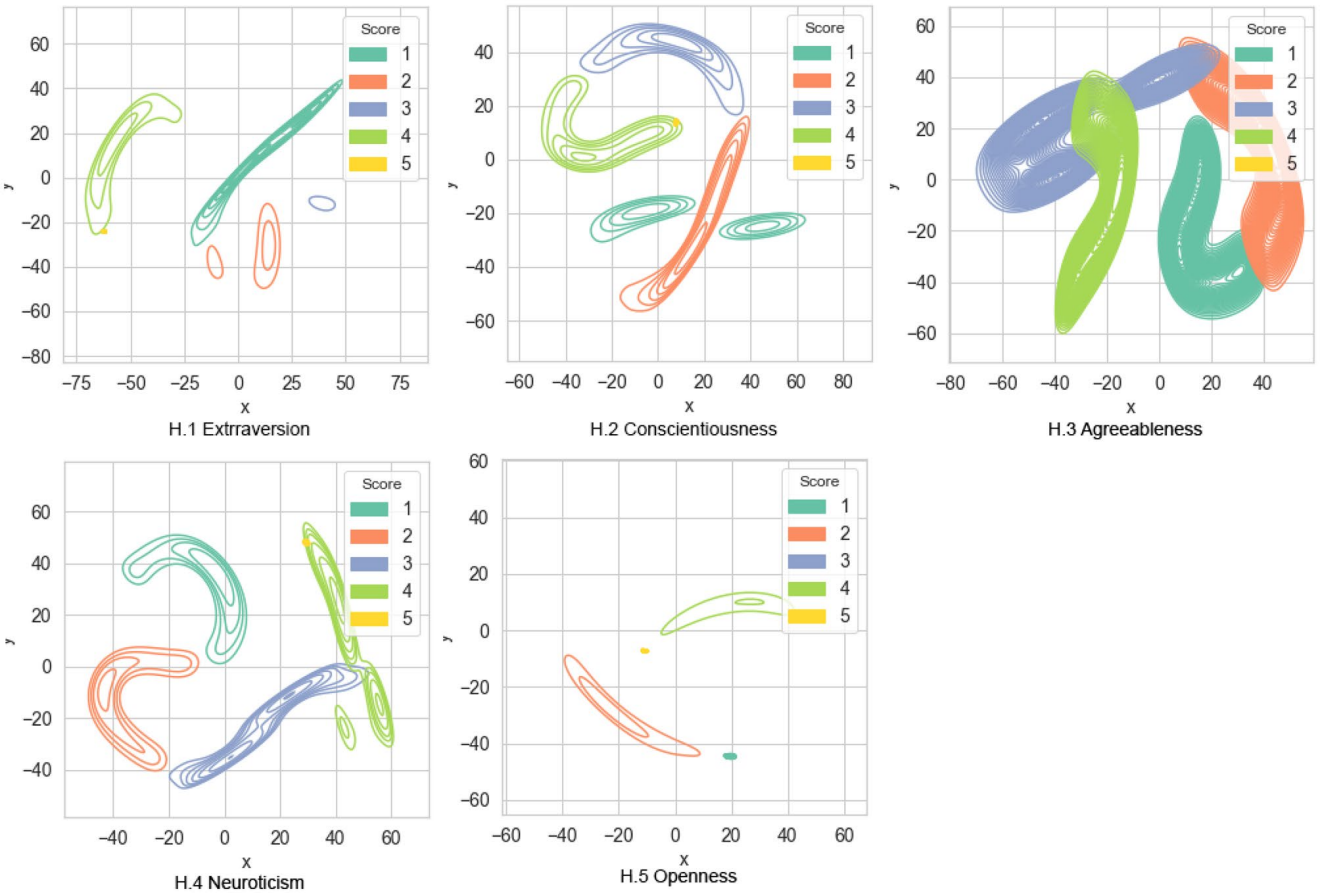
The clusters of feature impact assessments in the Big Five Trait diagnostic model using the Shapley Additive Explanation method.

Thus, the calculated Shapley values for each input example are subjected to further analysis according to the models corresponding to the assessed traits, taking into account the test subject's score obtained on the test scales. The distribution analysis of Shapley values provides insights into the stability of the assessed traits within the model across different severity categories. To achieve this, we round the subject's score to determine the category and assess the stability of feature effects. By clustering the obtained values and reducing dimensionality, we test the hypothesis that the resulting clusters correspond to the severity level of the assessed trait according to the scale score. Figure 13 (H) depicts the clustering results for Shapley values in the Big Five models.

In the clusters of coefficients corresponding to the *Extraversion* diagnostic model (H.1), it is worth noting the merging of clusters for scores 4 and 5, indicating the highest severity level of the assessed psychological property. This observation suggests a possible lack of differentiation in the test data for these two clusters. For the *Consciousness* diagnostic model (H.2), the Shapley values exhibit the merging of clusters for scores 4 and 5, along with partial intersection between clusters 1 and 2 with neighboring ones. Regarding the *Agreeableness* diagnostic model (H.3), the Shapley values demonstrate the allocation of clusters 1 and 2, representing low and below-average severity levels of the psychological trait, with an intersection observed in the threshold value area. Clusters corresponding to scores 3 and 4 exhibit some overlap. Cluster 5 for the maximum score on the scale is not explicitly expressed. Similar to Consciousness, the clustering of Shapley values for the Neuroticism diagnostic model (H.4) reveals a cluster structure with merged clusters for scores 4 and 5, as well as partial intersection among clusters representing neighboring severity levels. In the Openness diagnostic model (H.5), the clustering of Shapley values showcases the allocation of four distinct and non-overlapping clusters. However, the cluster corresponding to the mean severity level of the property (cluster 3) is not explicitly represented.

**Figure 14:**
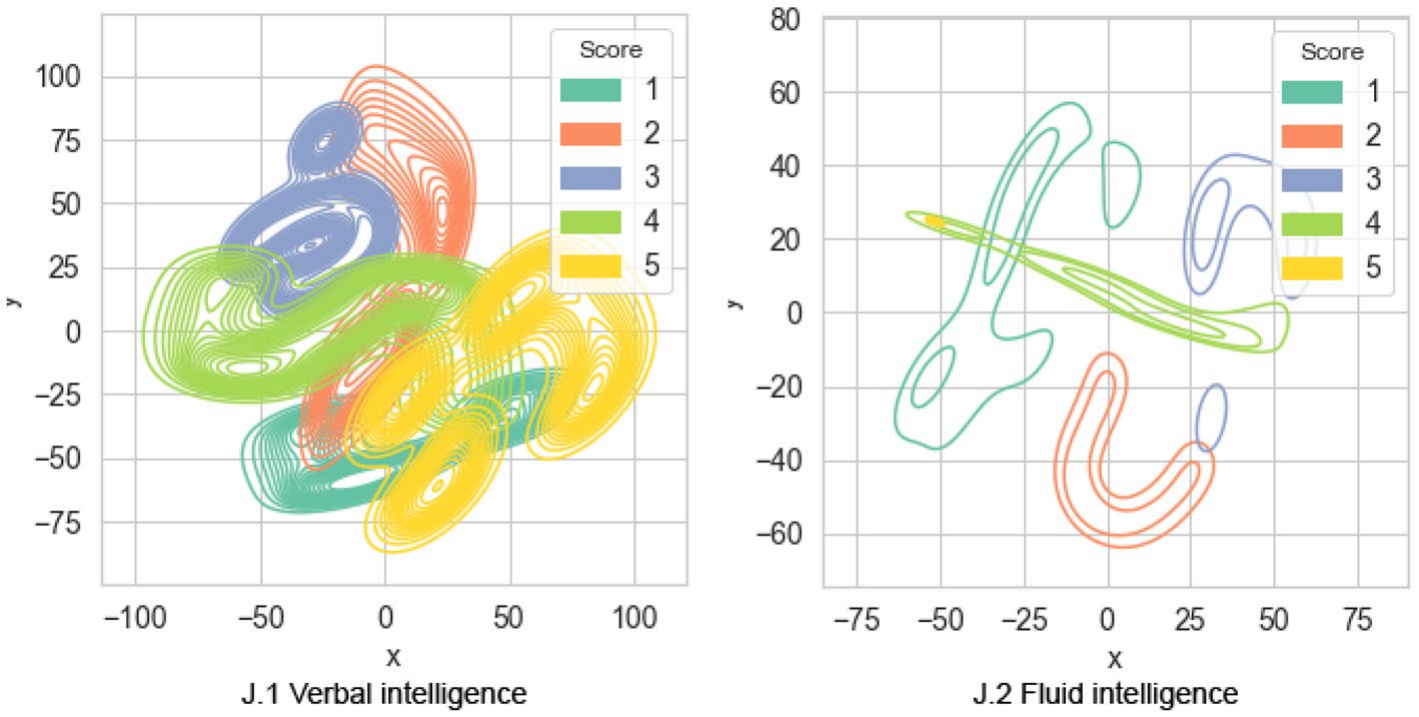
The clusters of assessments of the effects of traits in the model of intelligence diagnostics by the method of additive explanation of Shapley.

The results of Shapley values clustering for verbal and fluid intelligence diagnostic models are shown in Fig. 14 J. Clustering of the influence estimates of features obtained by the Shapley method in the model of *Verbal intelligence* diagnostics (J.1) does not manifest a clear separation of clusters. Cluster 5 is represented to a greater extent. Clusters 1, 2 and 3 have a significant intersection. Clustering of estimates of the features influence obtained by the Shepley method in the model of diagnostics of *Logical thinking* according to the Raven test (J.2) illustrates the possibility of identifying clusters. It should be noted that cluster 2 is represented, cluster 5 is included in cluster 4, and cluster 4 intersects clusters 1 and 3.

The conducted analysis demonstrates the potential for identifying clusters in the estimates of feature influence obtained using the additive explanation of Shapley. Notably, for several traits, particularly the scales of the Big Five test, we observe the emergence of clusters that align with the test subjects' scores. This consistency indicates the stable functioning of these traits and the extent of their influence on the model's predictions. However, in the model for diagnosing verbal abilities, the estimates of feature influence do not exhibit clear clustering. This lack of clustering may be attributed to the complex mechanism underlying the impact on the model's prediction outcomes for verbal abilities. Remarkably, the estimates of feature influence in the diagnostic model for Logical thinking display well-defined clusters for extremely high and extremely low values. This finding suggests that these extreme values have a significant impact on the model's predictions. Overall, it is important to highlight that the obtained results align with the accuracy of the model. The minimal prediction error is evidently manifested in the coordinated influence of features within the model.

## Discussion

The application of explainable AI-based approaches to diagnostic models of psychological properties and cognitive abilities has provided valuable insights into the contribution and mechanism of various signs. It should be noted that the findings have a peculiarity in terms of the Russian sample and the Russian social network, which forms a news feed according to certain algorithms, encouraging one or another content, thus setting the audience a certain vector of action. Therefore, the results discussed may differ from the results of other studies using the example of other social networks, but at the same time they can expand scientific knowledge in the field of the peculiarities referring to the manifestation of users’ behavioral and emotional characteristics on a social network.

Studying the characteristics of the features influence using various methods also involves analyzing their relationship with the value predicted by the model. Thus, clustering of the calculated coefficients of the used features influence in accordance with the degree of severity of the psychological property is another confirmation to the expediency of studying them as a complex. The analysis of the obtained clusters for the models indicates a pronounced differentiation of the indicator complex for the traits of the big five, depending on the degree of severity of the assessed property. For the verbal ability assessment model, the cluster distribution demonstrates the presence of possibly insignificant indicators that are not explicitly divided into 5 levels, but it can have an impact within adjacent levels, since a different combination of indicators has led to a significant decrease in the accuracy of the model.

The emotional coloring and tonality of the text are associated with such features as Extraversion, Openness to experience, Agreeableness, as they demonstrate the importance of the manifestation of the emotional properties referring to social network posts in the diagnosis of these properties, which is explained by the significance of their manifestation in behavior in real, rather than virtual life and the phenomenon of transferring the features of their offline behavior to online. Joy, Surprise and Anger stand out among the significant emotions. The significance of a number of emotional characteristics in intelligence diagnostic models seems ambiguous, which can be interpreted as the significance of the manifestation of the emotional sphere in the online activity of users with different levels of verbal and non-verbal abilities. Thus, a high level of Logical thinking is characterized by a low level of emotional expression of joy and surprise in social network posts. This result can be viewed through the prism of previously studied relationships between the level of cognitive abilities and psychological traits. Therefore, the demonstrated significant inverse correlation between the level of verbal intelligence and Agreeableness, Conscientiousness, as well as a significant direct correlation between the level of Logical thinking and Extraversion^55^ can also be identified and studied through the significance of the manifestation of certain emotional characteristics in the post during online activity on a social network. For example, the model for predicting the level of Logical thinking distinguishes the share of joyful reposts and share of posts with surprise among the significant features, as well as the model for diagnosing the level of Extraversion. The model of predicting the level of verbal abilities has shown the importance of two emotions—surprise and sadness, the latter of which intersects with the model of predicting the level of Agreeableness.

Analyzing significant indicators of user activity on a social network allows to study the features of user interaction with the audience and the functions of the social network, the use of which demonstrates their importance. The model for predicting the level of Logical thinking has shown the importance of a wide range of parameters, for example, indicators that characterize the interaction of a given user with other users on the network, their popularity (number of likes, comments and friends), and within the network functions is the number of videos, subscription to pages, and the total number of posts. A comparison of the revealed correlations between the indicators and the score predicted by the model provides a possibility to detect the multidirectional interest of users. The property of Openness to new experiences demonstrates an inverse correlation with the number of photo albums, whereas the analysis of the Extraversion diagnostic model, on the contrary, is direct. This means extroverts are more interested in broadcasting their life events to other users on the social network.

User activity indicators, particularly the frequency of posting material by day of the week, offer insights into the influence of weekends and working days. The placement of materials on a day off is significant for the properties of Consciousness, Extraversion, Neuroticism and Openness to new experiences and Logical thinking. The average number of publications on Monday is significant in the Benevolence diagnostic model. We can also observe cross-links between indicators. For example, a significant sign of activity in the model of predicting the level of Openness to experience is the total number of records, which is reflected in a consistent way by an inverse relationship with the average number of posts per day and a month from the group of frequency signs. To predict the level of Logical thinking, the significance of the total number of posts is expressed in a negative correlation with the average number of posts per day off.

In the group of the basic characteristics signs of posts and reposts, the highest absolute value of the average word length in posts is allocated for the model of predicting the verbal intelligence level, which is confirmed by the theory. The indicator of the average number of words in posts and reposts demonstrated an impact on predicting the level of Logical thinking. With regard to verbal intelligence, the model pays attention to the features of the words that the user types in, whereas for Logical thinking it is their number, which may be associated with building a chain of reasoning or a deeper and thoughtful expression of their thoughts.

The subject of the published posts is the most extensive category and can provide valuable information about areas of interest that may be significant in the diagnosis of psychological properties and cognitive abilities. The significance of the topic of crime, drugs, politics and religion in the model of predicting the level of Consciousness, where the placement of posts on the topic of religion has an inverse relationship and unlike the rest, can be interpreted from the point of view of the purpose of content placement. Highly conscientious people are not prone to provocation by others, respectively, the presence of these topics may indicate informing other users about certain events on these topics. Also, due to the fact that conscious people are attentive to controlling their behavior, they are not inclined to publish posts on religious topics, which may hurt the feelings of other people. On the contrary, friendly users tend to post a large number of such posts, which may be due to the cultural context, e.g. congratulating their friends and subscribers on social networks on religious holidays. There is also a low proportion of posts on the subject of escort, which defines them as users who are not inclined to condemn the lifestyle of other people. It is worth noting that the model for predicting the degree of Neuroticism covers a wide range of topics that are characterized by a reprehensible attitude of society, e.g. sexism, racism, pornography, which can be interpreted as difficulty in controlling an emotional state, instability of an emotional state, which can lead to intemperance and lack of compliance with social norms, as well as an emotional reaction to any events on the topics under consideration that cause a desire to share information with other network users.

Thus, we observe a logical transfer of offline behavioral features to a social network for a number of properties, however, for a number of properties we observe new trends in behavior in the online environment, which raises the question whether the opposite is true.

The construction of complex models with a large number of parameters, which certainly include neural networks, allows the model to take into account various relationships of the studied characteristics. The use of explicable AI methods makes it possible to open the “black box” and study the features of the signs in these models, gaining new knowledge about the direct manifestation of psychological and cognitive features in the online behavior of users. The proposed approach demonstrates the possibility of transition from studying the correlational relationships between various psychological properties to studying the similarity or difference of significant characteristics of online behavior in a social network, that are significant for diagnosing these properties through training neural network models and the use of explicable artificial intelligence methods.

## Material and methods

### The data

The sample of Russian-speaking users of the VK social network^49^ was 1358 people (46.7% male; M_age_ = 31.1) who underwent psychological testing and provided informed consent to access their VK social network profile data. 753,252 posts and reposts of these users were uploaded. Data collection of social network profiles and psychological diagnostics were carried out using a web service test.ipran.ru.

#### Ethics

The study was conducted in accordance with the ethical standards of the Russian Psychological Society, as well as the Helsinki Declaration of 1975 and its later amendments. Informed consent was obtained from all individual participants to be included in the study. The study was approved by the Ethics Committee of the Institute of Psychology of Russian Academy of Science (Ethic Committee approval code: 1-2023).

#### Psychological diagnostics

The main research hypothesis connects stable personal psychological traits of users with their behavior on the social network. One of the most popular models explaining the variability of feelings, behavior and thoughts is the Big Five model^56^. The stable orthogonal psychological traits that determine a person's characteristics include Big Five personal traits, designated as OCEAN (Openness to experience, Conscientiousness, Extraversion, Agreeableness, Neuroticism). To measure these traits, the Big Five Inventory questionnaire^50^ was developed in the Russian adaptation^57^.

Data on human behavior on social networks can be a new means of assessing human abilities, and therefore, within the framework of this study, the results of the crystallized and fluid intelligence assessment are analyzed. Crystallized intelligence is associated with the ability to rely on previously acquired knowledge. Basically, the level of development of this type of intelligence is assessed using verbal ability tests. In this work, we used a scale of analogies (20 tasks for 6 min) and generalizations (20 tasks for 7 min)^33^ and reasoning (16 tasks for 8 min)^58^. The overall score can be represented as the sum of the results on the scales. Fluid intelligence evaluates a person's ability to identify patterns, draw conclusions without relying on previous experience. This type of ability can be measured using the Raven's Advanced Progressive Matrices technique^19^. The tasks of the methodology are presented in the form of a 3 by 3 matrix consisting of geometric shapes, where one of the shapes is skipped and must be found among 8 alternatives. Thus, this technique evaluates the ability to inductive reasoning.

#### Social media data

The VK social network provides an opportunity for application developers to access user profile data using the application programming interface (API), which is a set of methods that return data in JSON format. According to the terms and regulations of the social network, to access the necessary user profile data, you must obtain an access key. This code becomes possible to obtain after the user's authorization on the social network on the page of an external Internet portal, which transmits a set of necessary attributes to the authorization widget, receiving an access key after authorization and granting permission by the user to access the data.

The VK social network API contains methods for accessing social network user profile data. Obtaining the values of various profile characteristics is possible after obtaining the informed consent of the user. The following profile parameters were highlighted: the count of friends, the count of subscribers, the count of photo albums, the count of photos, the count of audio recordings, the count of videos, the count of virtual gifts received, the count of communities, the count of objects in the “Interesting pages” block (bookmarks), age, education level, gender.

The indicated characteristics do not allow for analysis taking into account the dynamics of user activity on the social network. The social network does not contain information about changes in the count of friends, groups and other characteristics over time, however, we can access the user's posts on the wall in his profile on the social network, containing the attribute of the date and time of their placement, which allows to obtain the following data: date and time of posting, the text of the post, the text of the original in the case of a repost, the count of likes, the count of comments, the count of reposts, the count of views.

#### Features generation

The current level of development of natural language analysis models allows operating with a wide range of indicators for evaluating text characteristics. The language model BERT^59^, trained on the corpus of Russian-language texts^60^, allows to obtain various models of text classification on its basis. One of the earliest approaches is the sentiment analysis of the text, which provides the following assessments of the tonality of the text: negative, neutral, positive. With the widespread distribution of models of sentiment analysis of the text, the model for Russian language^61^ is used in this work. The next stage in the development of models is the training of the model for determining the emotional coloring of the text in 6 categories: joy, sadness, fear, surprise, anger, neutral text. In the current study the model^62^ trained on the corpus is used to obtain an estimate of the probability of the presence of each of the six analyzed emotions^63^. The practical application of natural language analysis models has found application in the field of identifying topics of user messages^64^, which allows identifying 18 topics^65^: gambling; pornography; prostitution; slavery, human trafficking, suicide: discussion of suicide methods; social injustice, inequality, social problems, class division of society; religion; terrorism; weapons; crime (murder, physical violence, child abduction), prison; online crimes: hacking passwords and accounts, viruses, pirated content, theft of personal information; politics, military service, past and present military conflicts; discussion of appearance, body, physical characteristics, appearance; discussion of health, physical and mental illnesses, disability; drugs, alcohol, smoking; racism and ethnicity; sexual minorities; sexism, gender stereotypes.

For further training of models, aggregated estimates are used for the proportion of posts and reposts of the user for each of the categories under consideration: sentiment analysis, emotional coloring of the text, thematic analysis, so that the sum of the values of the features for each of the categories according to the profile of one user is one.

Moreover, according to user posts and reposts, the following estimates of text properties are used: the share of posts in the total count of user posts; the share of reposts in the total count of user posts; the total count of user posts; the mean count of words in posts; the mean count of words in reposts; the mean length of words in posts; the mean length of words in reposts.

In terms of the frequency of content placement, the following activity indicators are generated: the mean count of messages per day, month, year; the mean count of messages by day of the week.

#### Profiles selection

A feature of any social network is the lack of sufficient activity of some users in the publication of posts, and, therefore, in the data obtained there are users whose activity is represented by listening to audio recordings and watching videos, materials published in user subscription groups, but there are the lack of publications that allow to apply text analysis methods that represent significant information for building models. As a criterion for selecting user profiles, the following are used: the presence of at least one user on the friends list; the presence of at least one post and a repost among the user's records; the presence of text data in posts and reposts.

The comparison of the results of constructing a model based on linear regression using the entire sample (indicators related to the texts evaluation of posts and reposts in profiles in which they are absent are 0) and profiles filtered according to the previously indicated principle (640 users social media profiles) demonstrate a significant increase in the explained percentage of variance (Table 1) using the example of the Big Five methodology.

**Table 1:** The comparison of the results of linear regression models for the entire sample (1358 profiles) and selected profiles (640 profiles).

Scale	R2 (all profiles)	R2 (selected profiles)
Neuroticism	0,09	0,21
Conscientiousness	0,08	0,18
Agreeableness	0,06	0,13
Extraversion	0,08	0,17
Openness to experience	0,08	0,16

Thus, users who have a social network profile that they use to read the news feed, posts in the groups, listen to music and watch videos, but do not publish any records (posts and reposts) on social networks, do not provide sufficient information for the possible construction of a model for the psychological traits diagnosis. Further work with user activity data in the VK social network is carried out taking into account the criteria of user activity for posts and reposts.

#### Feature selection

User activity data on the social network have different scales and are represented by fractions, absolute and mean values of indicators. Thus, further work with data is possible through standardization or data scaling. The MinMaxScaler method allows you to scale data to a range of 0–1, which allows to identify features in which the smallest spread is observed, which does not represent meaningful data for analysis. By setting a threshold of 0.002 for the 25th percentile and 0.01 for the 75th percentile, we receive the opportunity to select features containing a spread sufficient for training a neural network. However, the presence of a sufficient spread of the values of the analyzed indicators in the sample as a whole does not mean that for subjects with varying degrees of severity of the assessed psychological trait that there will also be a sufficient spread of indicators, as a result of which the selection procedure for signs looks as shown in Supplementary Algorithm S1.

As a result, for each of the methods used, sets of features containing a sufficient spread were obtained. For the data set of the results of the "Big Five", "Logical thinking " tests (the "Advanced Progressive Raven Matrices" techniques), 76 characteristics were selected during the analysis, and 58 signs were selected for the verbal abilities diagnostic test (information about the characteristics included in each of the models is contained in Supplementary Table S1).

#### Data augmentation

The approach using data augmentation is described for various machine learning tasks: speech recognition^66^, data segmentation^67^, image recognition using GAN^68^ and clearing part of the image^69^, natural language analysis^70^, time series predictions^71^, as well as an integrated approach using normalization and data generation^42^. In this paper, as an approach to data augmentation, a method based on the characteristics of the distribution of signs in the profiles of testees with varying degrees of severity of psychological properties is used. The data augmentation procedure is presented Supplementary Algorithm S2.

The application of this approach allows the trained neural network to increase the generalizing capability and overcome the problem of retraining, which is relevant for a small sample size. Prior to the start of the augmentation procedure, a control sample is formed, which makes up 12% of the records that are not involved in the procedure of generating and further training the model.

Thus, separate samples were formed for each of the scales of psychodiagnostic techniques used: training, test and control. The training and test samples were randomly formed from combining selected profiles and augmented data, and the control sample from selected profiles, which were not participating in augmentation.

### Modeling approach

**Figure 15:**
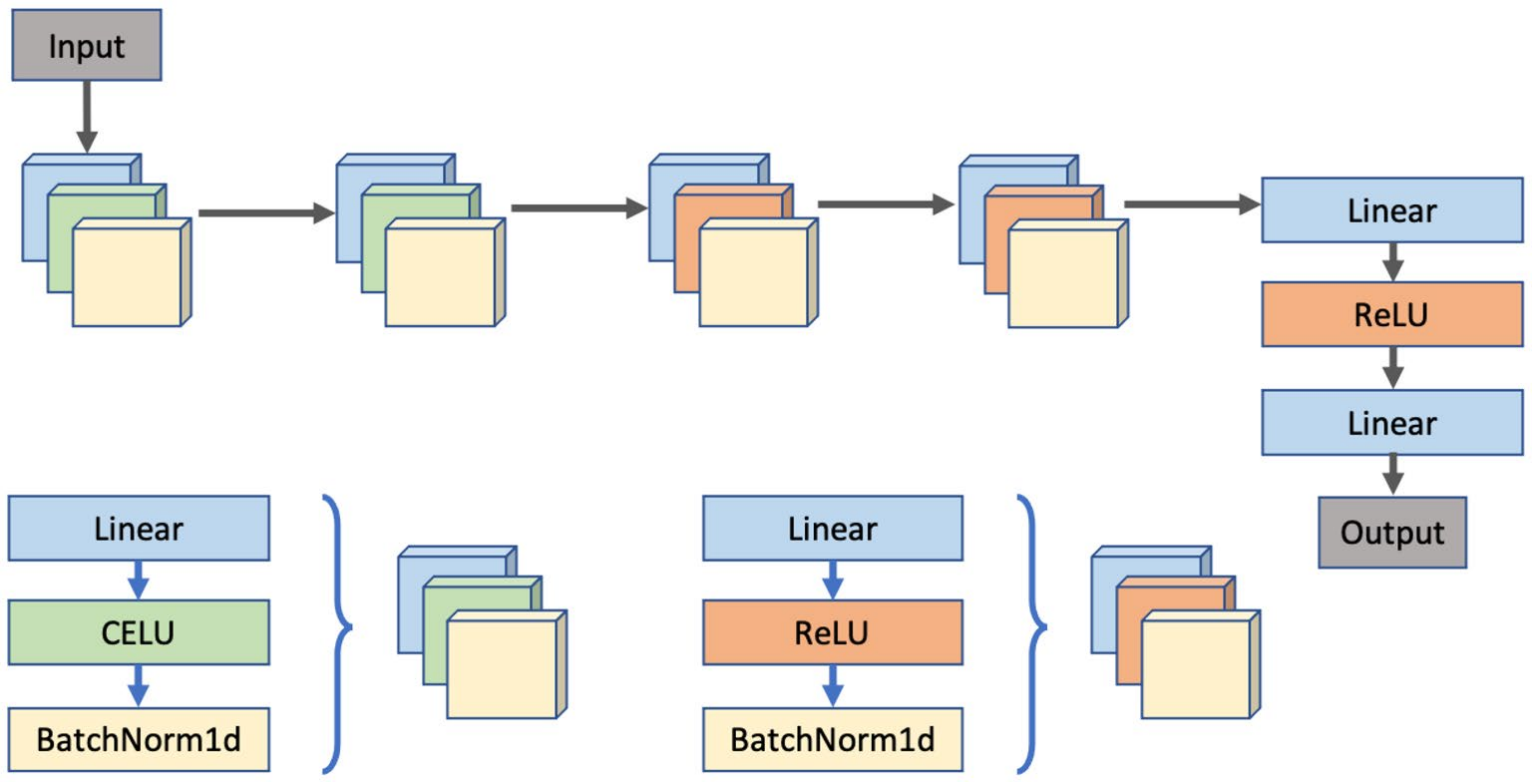
The structure of the model.

The model for predicting psychological traits receives an input vector of signs of user activity in the VK social network with a size of 68 (the size of the input layer depends on the psychological test) elements and is represented by a sequence of seven fully connected Linear layers with a size of 68 × 30, 30 × 25, 25 × 20, 20 × 10, 10 × 5, 5 × 3, 3 × 1. The Celu activation functions are applied to the first three Linear layers^72^, and the ReLU activation function is applied to the fourth, fifth and sixth Linear layers^73^. The first five layers with activation functions are followed by feature normalization layers (BatchNorm1d). The description of the model layers is presented in Figure 15.

The model is implemented using the PyTorch library^74^ and trained at a batch size of 64 using an optimizer based on Stochastic Gradient Descent^75,76^, and a learning rate of 0.001 per 1000 epochs. The best model is determined by the minimum value of the mean squared error on the test and control samples.

The learning results (R^2^—coefficient of determination; MSE—mean squared error) are given in Supplementary Table S2, where the highest accuracy for the model of determining the level of Extraversion among the Big Five traits and verbal abilities for intelligence models can be seen.

### Explainable AI methods

**Figure 16:**
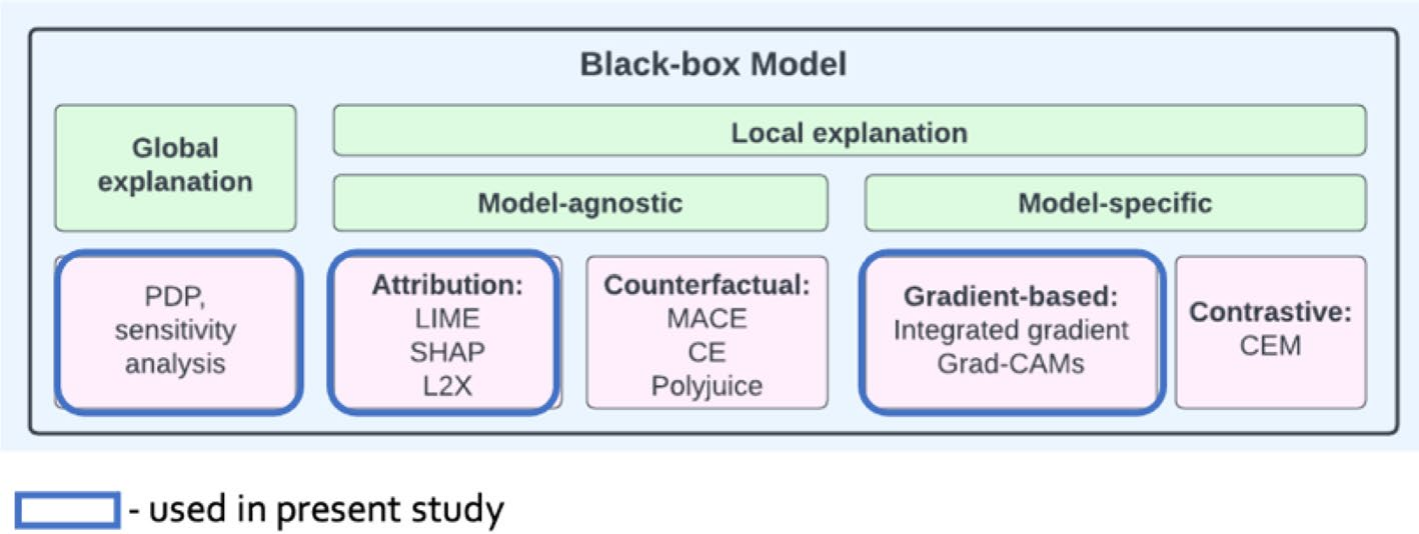
Family of explanation methods.

A number of tools created in recent years offer the implementation of various approaches to the problem of interpreting the work of neural network models. Figure 16 presents a classification^77^ of existing approaches and highlights the sections used in current study.

The Captum^53^ library contains the implementation of a count of algorithms based on gradients that determine the importance of features, neurons and layers of the trained network, as well as a set of indicators for evaluating the performance of these algorithms. This approach can be used for neural network classification and regression models using different input data modality: images, tabular data, text. Within the framework of this work, signs are selected for analysis, the loads of which fall into the 5th and 95th percentiles, that is, they fall into 5% of the maximum and minimum values in the sample containing estimates of the influence of signs for the model obtained by the Integrated gradients method. Such an approach can demonstrate the maximum positive value for one trait obtained by one of the methods and the maximum negative value obtained by another method, which on the one hand indicates the high significance of the trait, on the other hand, does not allow assessing the direction of the influence of the trait. This approach allows us to obtain estimates of the influence of features for one input observation, which lets us analyze the features of the model for user profiles with high, medium and low values on the scale of psychological methodology. The PDPbox^54^ library provides an opportunity to evaluate the relationship of a trait and model predictions for different intervals of this trait (we calculated correlation estimates between the value of each feature and the value predicted by the model). The use of a set of these methods makes it possible to consider the features of the work of trained models from different sides. The SHAP^78^ library is based on assigning each feature an important indicator for each forecast, calculating the impact of the feature based on its changes in the dataset and the corresponding changes in the model forecast. Further work with the calculated Shapley values is associated with clustering by the K-Means^79^ method and dimensionality reduction using the t-distributed Stochastic Neighbor Embedding (t-SNE)^80^ algorithm.

### Supplementary Information

Supplementary Information.

## Data Availability

The study materials and data can be accessed at https://ieee-dataport.org/documents/social-network-dataset-vkontakte-users-profile-psychological-characteristics-verbal-and.
