# Supplementary material for: Applying explainable artificial intelligence methods to models for diagnosing personal traits and cognitive abilities by social network data
Source: Sci Rep. 2024 Mar 4;14:5369. doi: 10.1038/s41598-024-56080-8 (PMC10912674; doi:10.1038/s41598-024-56080-8)

# **Supplementary information**

## **Table S1**. Distribution of significant features of user activity in the social network VKontakte in models

| **Feature** | **Big Five** | **Verbal intelligence** | **Fluid intelligence** |
| --- | --- | --- | --- |
| **Activity** | **17** | **15** | **17** |
| albums | 1 | 1 | 1 |
| audios | 1 | 1 | 1 |
| comments | 1 | 1 | 1 |
| count_mess | 1 | 1 | 1 |
| followers | 1 | 1 | 1 |
| gifts | 1 | 1 | 1 |
| groups | 1 | 1 | 1 |
| likes | 1 | 1 | 1 |
| pages | 1 | 1 | 1 |
| photos | 1 |  | 1 |
| post | 1 | 1 | 1 |
| repost | 1 | 1 | 1 |
| reposts | 1 | 1 | 1 |
| subscriptions | 1 |  | 1 |
| videos | 1 | 1 | 1 |
| views | 1 | 1 | 1 |
| friends | 1 | 1 | 1 |
| **Person characteristic** | **2** | **2** | **2** |
| age | 1 | 1 | 1 |
| education | 1 | 1 | 1 |
| **Text characteristics** | **4** | **4** | **4** |
| count_words_post | 1 | 1 | 1 |
| count_words_repost | 1 | 1 | 1 |
| mean_words_len_post | 1 | 1 | 1 |
| mean_words_len_repost | 1 | 1 | 1 |
| **Theme** | **28** | **15** | **28** |
| post_theme_body_shaming | 1 | 1 | 1 |
| post_theme_drugs | 1 |  | 1 |
| post_theme_health_shaming | 1 | 1 | 1 |
| post_theme_offline_crime | 1 | 1 | 1 |
| post_theme_online_crime | 1 |  | 1 |
| post_theme_politics | 1 |  | 1 |
| post_theme_pornography | 1 | 1 | 1 |
| post_theme_prostitution | 1 |  | 1 |
| post_theme_racism | 1 |  | 1 |
| post_theme_religion | 1 |  | 1 |
| post_theme_sexism | 1 |  | 1 |
| post_theme_social_injustice | 1 | 1 | 1 |
| post_theme_weapons | 1 |  | 1 |
| repost_theme_body_shaming | 1 | 1 | 1 |
| repost_theme_drugs | 1 | 1 | 1 |
| repost_theme_health_shaming | 1 | 1 | 1 |
| repost_theme_offline_crime | 1 | 1 | 1 |
| repost_theme_online_crime | 1 | 1 | 1 |
| repost_theme_politics | 1 | 1 | 1 |
| repost_theme_pornography | 1 | 1 | 1 |
| repost_theme_prostitution | 1 |  | 1 |
| repost_theme_racism | 1 |  | 1 |
| repost_theme_religion | 1 | 1 | 1 |
| repost_theme_sexism | 1 | 1 | 1 |
| repost_theme_sexual_minorities | 1 |  | 1 |
| repost_theme_social_injustice | 1 |  | 1 |
| repost_theme_suicide | 1 |  | 1 |
| repost_theme_weapons | 1 | 1 | 1 |
| **Sentiment** | **3** | **3** | **3** |
| negative | 1 | 1 | 1 |
| neutral | 1 | 1 | 1 |
| positive | 1 | 1 | 1 |
| **Frequency** | **10** | **9** | **10** |
| day_mean | 1 | 1 | 1 |
| Fri | 1 | 1 | 1 |
| Mon | 1 | 1 | 1 |
| month_mean | 1 | 1 | 1 |
| Sat | 1 | 1 | 1 |
| Sun | 1 | 1 | 1 |
| Thu | 1 | 1 | 1 |
| Tue | 1 | 1 | 1 |
| Wed | 1 | 1 | 1 |
| year_mean | 1 |  | 1 |
| **Emotional evaluation** | **12** | **10** | **12** |
| anger | 1 | 1 | 1 |
| anger_repost | 1 | 1 | 1 |
| fear | 1 |  | 1 |
| fear_repost | 1 |  | 1 |
| joy | 1 | 1 | 1 |
| joy_repost | 1 | 1 | 1 |
| no_emotion | 1 | 1 | 1 |
| no_emotion_repost | 1 | 1 | 1 |
| sadness | 1 | 1 | 1 |
| sadness_repost | 1 | 1 | 1 |
| surprise | 1 | 1 | 1 |
| surprise_repost | 1 | 1 | 1 |
| Total | 76 | 58 | 76 |

**Table S2.** Models accuracy for Big Five traits and cognitive abilities.

| Sample | R^2^ | MSE |
| --- | --- | --- |
| **Neuroticism** | | |
| Train | 0,97 | 0,03 |
| Test | 0,96 | 0,04 |
| Valid | 0,35 | 0,19 |
| **Extraversion** | | |
| Train | 0,99 | 0,01 |
| Test | 0,98 | 0,02 |
| Valid | 0,44 | 0,16 |
| **Conscientiousness** | | |
| Train | 0,99 | 0,01 |
| Test | 0,98 | 0,02 |
| Valid | 0,42 | 0,15 |
| **Agreeableness** |  |  |
| Train | 0,99 | 0,002 |
| Test | 0,99 | 0,01 |
| Valid | 0,39 | 0,17 |
| **Openness** | | |
| Train | 0,99 | 0,009 |
| Test | 0,99 | 0,01 |
| Valid | 0,43 | 0,11 |
| **Verbal intelligence** | | |
| Train | 0,98 | 0,02 |
| Test | 0,97 | 0,03 |
| Valid | 0,49 | 0,50 |
| **Fluid intelligence** | | |
| Train | 0,99 | 0,01 |
| Test | 0,99 | 0,01 |
| Valid | 0,38 | 0,6 |

**Algorithm S1:** Feature selection procedure for one scale


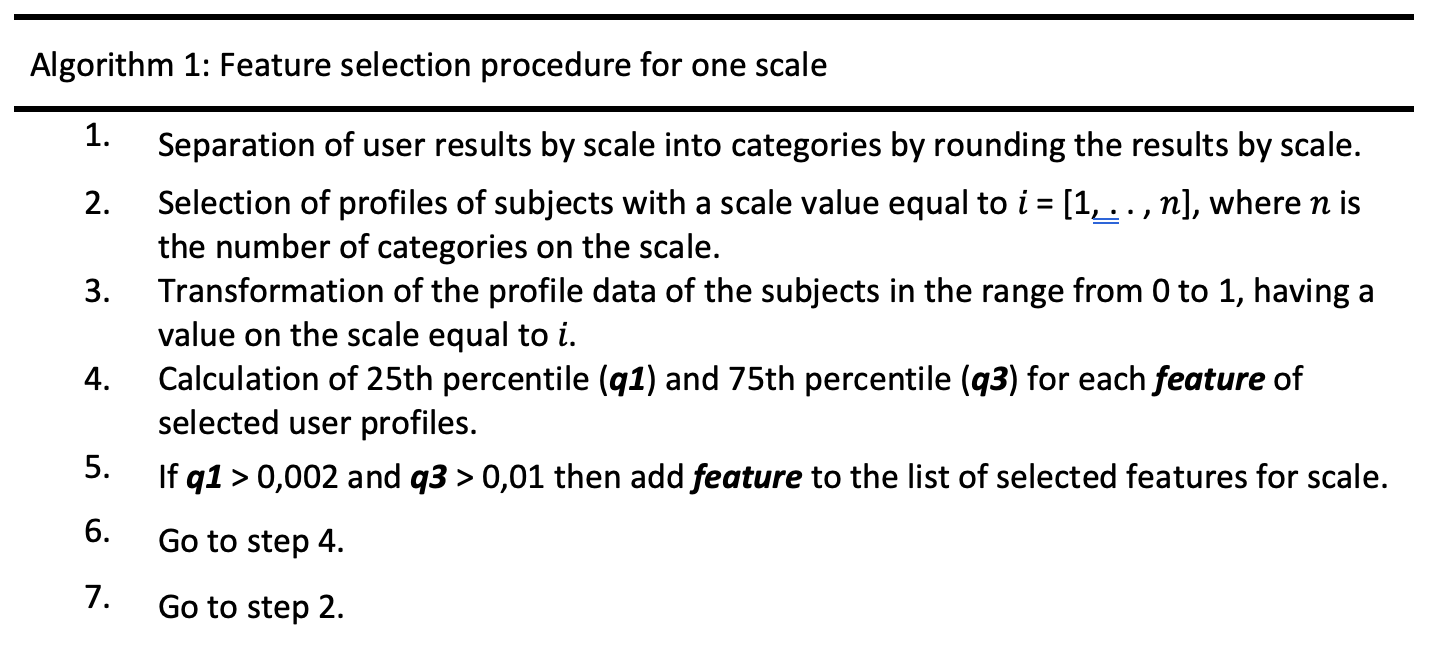


**Algorithm S2:** Data augmentation procedure for one scale


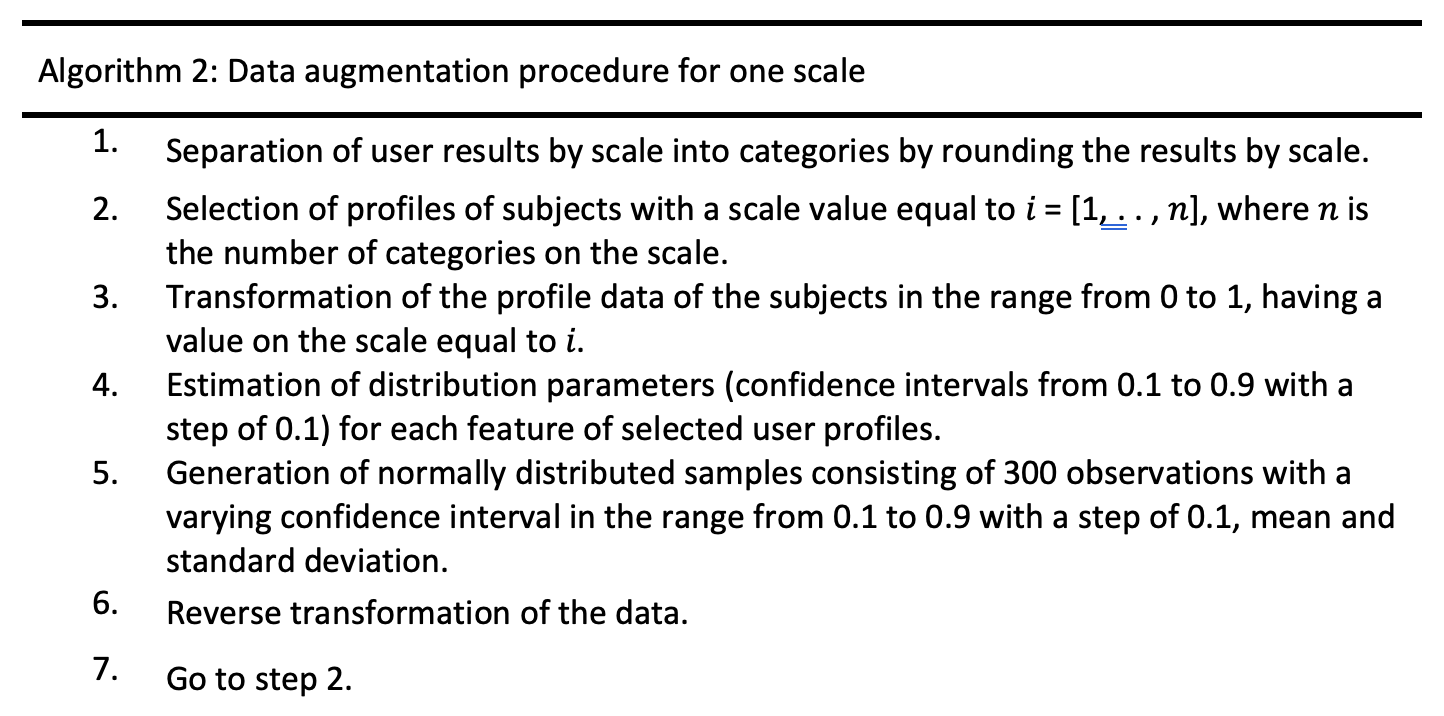

Supplement: Supplementary file 1 — Supplementary Information. [file 41598_2024_56080_MOESM1_ESM.docx]
